# Lightweight Detection of Inserted Chirp Symbols in Radio Transmission from Commercial UAVs

**DOI:** 10.3390/s25154552

**Published:** 2025-07-23

**Authors:** Krzysztof K. Cwalina, Piotr Rajchowski, Jarosław Sadowski

**Affiliations:** Faculty of Electronics, Telecommunications and Informatics, Gdansk University of Technology, 80-233 Gdansk, Poland; krzysztof.cwalina@pg.edu.pl (K.K.C.); piotr.rajchowski@pg.edu.pl (P.R.)

**Keywords:** chirp, drone, lightweight detection, RF signal detection, unmanned aerial vehicle, UAV

## Abstract

Most small, commercial unmanned aerial vehicles (UAVs) maintain continuous two-way radio communication with the controller. Signals emitted by the UAVs can be used for detection of their presence, but as these drones use unlicensed frequency bands that are shared with many other wireless communication devices, UAV detection should rely on the unique characteristics of the transmitted signals. In this article, low-complexity methods for the detection of chirp symbols in downlink transmission from a UAV produced by DJI are proposed. The presented methods were developed with focus on the ability to detect presence of chirp symbols in radio transmission without a priori knowledge or need for center frequency estimation.

## 1. Introduction

The development of technology for the production of electric motors, batteries, and control and communication systems has allowed common use of unmanned aerial vehicles (UAVs) in various applications in recent years. Autonomous or remotely controlled drones, which 20 years ago were used only in professional applications due to their high cost and complicated use, nowadays are freely available at affordable prices, which allows them to extend their possible usage to fields such as entertainment [1], environment monitoring [2,3,4], mapping [5,6], disaster management [7], search and rescue missions, inspections of infrastructure [8], or even agriculture [9,10]. The wide availability of UAVs is also one of the causes of the observed growth in malicious activities. There are many examples of disturbances in air traffic and even shutting down airports due to observed presence of unauthorized and unidentified remotely controlled drones in controlled airspace [11,12,13]. Unmanned aerial vehicles may be used to invade privacy and security by taking pictures and movies of people not obeying their rights, by filming strategic areas and objects such as power plants or sea ports, by smuggling illegal cargo, etc. [11]. The presence of unauthorized drones may also impact technological processes in factories and in case of drone failure, uncontrolled flight or deliberate action drones can cause failures and downtimes and even disasters. For these reasons, in many cases, it becomes necessary to use systems and devices that can warn about the presence of unauthorized drones in the vicinity of a given facility.

Many commercial UAVs use unlicensed Industrial, Scientific, and Medical (ISM) bands for both control (in uplink) and video data streaming with telemetry (in downlink). The most commonly used frequency ranges are 2.4–2.483 GHz and 5.15–5.9 GHz. The same parts of the spectrum are used for short-range wireless communication in form of wireless local area networks (WLANs, e.g., WiFi) and wireless personal area networks (WPANs, e.g., Bluetooth). Therefore, in urban areas, these ISM bands are very intensively used for different purposes. Consequently, the detection of UAV presence and communication in 2.4 GHz or 5 GHz bands based on the simple detection of radio signal energy is highly unreliable as it may frequently trigger false alarms caused by WLAN and WPAN emissions.

A review of drone detection and identification methods based on the reception of radio signals transmitted by UAVs can be found, e.g., in work by Jurn et al. [14]. The majority of UAV detection methods mentioned in this review use the characteristic parameters of received signals, called RF (radio frequency) fingerprints, or the coherent reception of signals by antenna arrays of various design. Such antenna arrays not only can distinguish intentional emission from random noise but can also detect the angle of signal arrival that can be used to estimate UAV position or at least bearing.

An important group of solutions designed to detect and sometimes also classify drones utilizes various implementations of neural networks. In [15], samples of radio signals from a UAV were converted into spectrum charts or spectrograms and then processed using neural networks as typical images. The review article [11] presents several solutions that utilize AI-based RF signal processing for drone detection, including multi-stage detection and classification of UAV type (producer) and even mode of flight. However, the large group of solutions presented in [11] was developed and tested only using the UAV RF signal dataset “DroneRF” [16], which was prepared using a setup with two software-defined radio (SDR) receivers, each of them covering only half of the recorded signal bandwidth [17]. Another, different dataset, “CardRF” [18], was used in research works described in [19] to train a multilayer convolutional neural network using deep learning algorithms that achieved over 97% accuracy in the separation of UAV signals from other signals present in the 2.4 GHz band (WiFi, Bluetooth) and 84% accuracy in RF signature classification. Neural networks and deep learning techniques can also support audio-based drone detection. Results presented in [20] show that convolutional neural networks (CNNs) not only can detect some small commercial drones but also classify them using audio signatures with over 92% accuracy.

The presence of UAVs can be detected not only by the reception of RF signals. Thermal detection using infrared cameras and analysis of images from visible light cameras is mentioned in [13,21], together with acoustic detection and radar-based solutions. These non-RF detection methods can be especially useful in case of drones which are not controlled in real time and are not transmitting real-time video or other data. However, considering low-cost commercial quadcopters, which constitute the majority of UAVs in private use, their abilities to perform autonomous missions without real-time control transmission are limited. Therefore, detecting the presence of privately owned UAVs that may violate the privacy of individuals may be based on the RF emission from both the UAV and controller.

A high efficiency of UAV detection can be obtained by using full decoding of RF communication protocol or traffic analysis [22]. Nevertheless, modern commercial UAVs can use more complex and advanced communication protocols [23] like LightBridge or MAVLink [24]. This results in a different pattern of transmitted data packets, compared to Wi-Fi, as well as for communication and video transmission. Modifications introduced in subsequent revisions extend the range of possible configurations, like the packet length or packet transmission periodicity [23]. Another method of UAV detection may combine the protocol analysis on the level of transmitted repetitive data bytes [25]. The UAV’s control or data packets may include characteristics fields, like user or command IDs, which simplifies the protocol pattern and minimizes the statistical analysis [25].

Unfortunately, in some cases, decoding the radio communication transmitted even in the unlicensed band (ISM) but not addressed to the person or entity that uses drone detection devices may be treated as unauthorized access to correspondence. This method of detecting UAVs used by private users and not by authorized entities may constitute a violation of the law. To avoid such unclear situations, UAV communication detection methods presented in this publication are based only on detection the presence of characteristic signals with linear frequency sweep (chirp signals) in radio emissions from commercial UAVs produced by DJI company (Shenzhen Da-Jing Innovations Sciences and Technologies Ltd., Nanshan District, Shenzhen, China). The vast majority of drones from this manufacturer use the OcuSync radio communication protocol based on the orthogonal frequency division multiplexing (OFDM) technique with Zadoff–Chu sequences for synchronization and/or channel parameter estimation [26,27], but at least part of Zadoff–Chu symbols are characterized by linear frequency sweep and can be processed in the physical layer as chirp symbols without the need to implement full packet decoding. These signals do not carry any information as they are most probably used for channel sounding and estimation of radio communication quality; therefore, the reception and detection of chirp signals cannot be treated as unauthorized access to any kind of information. A unique feature of the proposed chirp signal detection methods is the ability to work independently to the shift in the center frequency of the received UAV’s signal, as long as whole signal spectrum fits into the receiver bandwidth.

## 2. Problem Formulation

**Figure 1:**
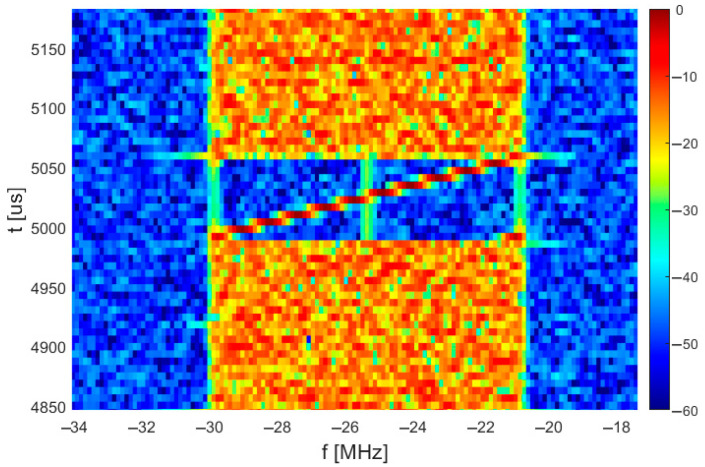
Single chirp-like symbol in spectrogram of DJI Matrice 300 downlink signal.

Most small commercial UAVs for personal use transmit video stream and sideband telemetry data in wideband emission (a few to over a dozen MHz of bandwidth) in one of the ISM bands: 2.4 GHz or 5 GHz. The vast majority of these emissions is based on orthogonal frequency division multiplexing (OFDM) signals or at least signals that show the most characteristic property of OFDM: cyclic prefix. Unfortunately, the OFDM technique is also used in the physical layer of radio communication networks like WiFi, which allows them to achieve high data rates. Consequently, simple detection based on periodic autocorrelation including the cyclic prefix may be used to decide whether the ISM band is used for wideband communication, but it usually cannot be used to distinguish communication patterns between UAVs and WiFi. However, during an analysis of signals transmitted by several small commercial drones, it was discovered that the digital video stream transmitted by UAVs produced by DJI contains symbols that look like “chirp” symbols with linear sweep of instantaneous frequency (Figure 1). The presented examples show signals from “Matrice 300”, which is an industrial-grade drone produced by DJI, but the same signal structure and identical chirp symbols are also present in signals emitted by other UAVs from the same manufacturer that implement the OcuSync radio communication protocol.

Chirp signals are usually used in applications related to precise signal timing measurements, such as radars [28], radio altimeters [29] and devices which provide measurements of distance between nodes in radio networks. The chirp symbols in video streams from UAVs from DJI are transmitted periodically every 20 ms, but their parameters are not always the same. The downlink transmission bandwidth is variable and depends on both the UAV model and current channel state as the physical layer link parameters (carrier frequency, bandwidth) are variable and adaptive. In the case of downlink emission with a bandwidth of approx. 9 MHz and 18 MHz, a single up-chirp symbol is observed with comparable duration time (66 μs, exact value cannot be precisely estimated), which occupies the whole emission bandwidth; therefore, the frequency slope rate in case of a larger bandwidth is higher. However, in the case of a 36 MHz emission bandwidth, it was estimated that two symbols are transmitted simultaneously in 18 MHz wide subchannels. Moreover, sometimes, a kind of wideband irregular signals are transmitted by UAVs with a constant bandwidth of 9 MHz. In case of these emissions, other chirp-like signals are observed but with reversed direction of frequency change (down-chirp), which can sometimes be transmitted twice, immediately one after another.

**Figure 2:**
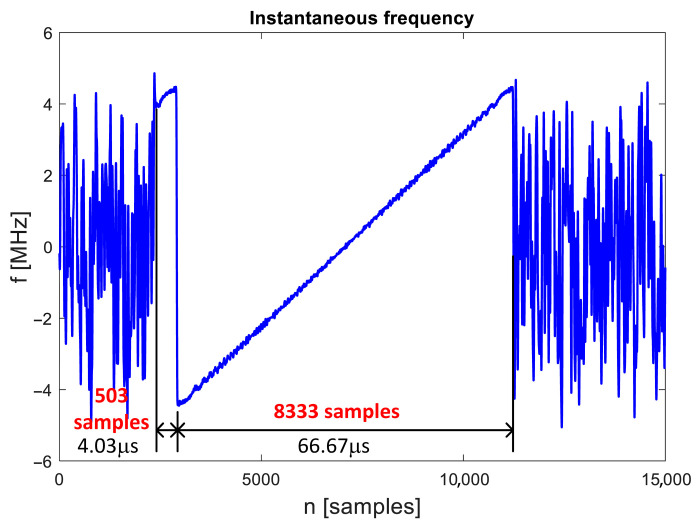
Instantaneous frequency of UAV downlink signal in chirp symbol. UAV model: DJI Matrice 300, 9 MHz transmission bandwidth, signal sampling rate: 125 MHz.

Fast Fourier Transform (FFT)-based spectrograms present signal power distribution in time and frequency where both the time and the frequency domain is discrete. Therefore, Figure 1 does not prove that analyzed signals really contain symbols with linear frequency change in time (both domains are continuous), because similar charts can be obtained e.g., from series of short symbols with constant carrier frequency. To verify the signal characteristics, the instantaneous signal frequency was calculated as a derivative of the signal phase. An example plot of instantaneous frequency is presented in Figure 2. It proves that the observed frequency change is linear and continuous inside a symbol duration of approx. 66.67 μs. Unexpectedly, before the main part of the chirp symbol, during a time period of slightly more than 4 μs (precise estimation of symbol time is not possible from single symbol analysis), the signal frequency is the same as in the last part of the main symbol. It may be caused by the generation of a whole signal, both OFDM part and chirp symbols (OFDM symbols with Zadoff–Chu sequences), by the same transmitter software that adds cyclic prefix to all processed symbols. The cyclic prefix is meaningless for chirp detection as there is no signal frequency/phase continuity between the end of the cyclic prefix and the beginning of the main part of the chirp symbol, and it probably cannot be used for chirp symbol detection.

The frequency change rate in the chirp symbols was estimated by evaluating the parameters of linear approximation of instantaneous frequency. In the case of a 9 MHz signal bandwidth (signal type 1), the change rate is approx. +135.2 kHz/μs, while the value obtained for an 18 MHz signal is almost exactly two times higher: +270.2 kHz/μs. The down-chirp symbols have a frequency change rate of approx. −135.3 kHz/μs. The instantaneous frequency method cannot be used to estimate the signal change rate in case of an emission bandwidth 36 MHz, but considering the observed spectrograms, one can expect that the signal frequency change rate in the case of 36 MHz emission will be the same as in the case of 18 MHz signal bandwidth.

The goal of the presented research works was to find a low-complexity method for chirp (OFDM symbols with Zadoff–Chu sequences) detection in radio communication with DJI UAVs, although in the case of video stream transmission in downlink, chirp symbols are observed regularly with repetition period equal 20 ms. Despite that phenomenon, a simple autocorrelation of UAV communication signals with 20 ms delay such as

(1) $$R_{20}[t]=\frac{\sum_{\tau =0}^{66\mu s}x[t+\tau ]\cdot x^{*}\left[t+\tau +20\;ms\right]}{\sqrt{\sum_{\tau =0}^{66\mu s}{\left|x\left[t+\tau \right]\right|}^{2}}\cdot \sqrt{\sum_{\tau =0}^{66\mu s}{\left|x\left[t+\tau +20\;ms\right]\right|}^{2}}}$$

cannot be used for reliable chirp symbol detection because other fragments of UAV downlink emission, which are most probably video stream packets and telemetry transmissions using the OFDM technique, can also contain highly repeatable parts with autocorrelation properties comparable to a case of chirp emission. The time domain in (1) is discrete, as all signal processing was made using a stream of samples from a universal software radio peripheral (USRP) front end, but to obtain a general form of equations regardless of the signal sampling rate, the time is expressed in seconds, not in sample numbers.

**Figure 3:**
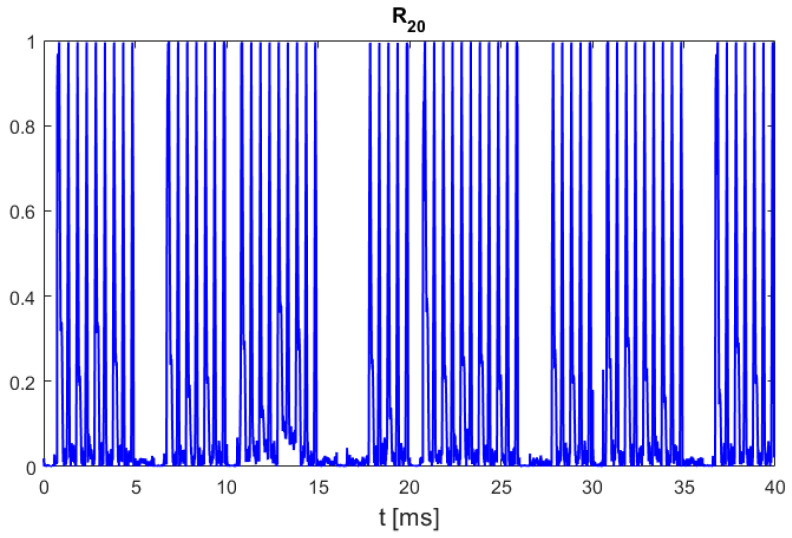
Normalized autocorrelation of UAV emission with 20 ms delay, correlated signal duration: 66 μs. UAV model: DJI AIR 2S, 18 MHz signal bandwidth.

The plot in Figure 3 presents a normalized autocorrelation (1) of a 40 ms long UAV emission fragment (18 MHz bandwidth) that contains two chirp symbols. In this example, analysis was performed on signals from an “AIR 2S” drone produced by DJI, which is a small, low-cost UAV for private use. Unfortunately, the presence and position of chirp symbols is not clearly indicated here as the same value of (1) is also obtained for other repetitive but non-chirp signal fragments. But it must be emphasized that, in contrast to repetitive chirp symbol emission, the presence of other fragments with 20 ms periodicity in UAV downlink signals is variable and not always observed.

Furthermore, such a signal autocorrelation method cannot be considered as suitable to detect short-time emission that was classified as channel sounding signals, because in most cases, such signals that contain single or double down-chirp symbols are transmitted irregularly, with highly probable single emissions and emissions on variable carrier frequency. Therefore, another method for chirp symbol detection was needed, with the ability to detect single symbols in short transmissions.

## 3. Chirp Symbol Detection Methods

The most obvious method for chirp symbol detection, which should provide the best detection quality, is cross-correlation with a locally generated chirp template (reference chirp signal) or, equivalently, a matched filter. Unfortunately, in addition to the need for knowledge of the exact shape of the symbol to be detected, both cross-correlation and matched filtering also require the signal center frequency to be known and fixed, while real UAV emission carrier frequency is variable and random. Compensation for variable carrier frequency requires earlier detection of the UAV’s signal frequency using other methods, which makes further chirp symbol detection pointless. Thus, three methods for UAV signal detection focusing on detecting chirp symbol properties without need for knowledge of the signal carrier frequency are presented in the following subchapters.

### 3.1. Method I

The first method is based on correlation of received chirp-like symbols with locally generated reference signal with linear change of instantaneous frequency. But compared to cross-correlation, which is equivalent to matched filtering, in which the start frequency, stop frequency, and frequency change rate must correspond to chirp symbol parameters, the instantaneous frequency of the template signal is swept in the whole range defined by the signal sampling rate $f_{s}$ or the boundaries of the monitored frequency range. A template chirp signal that has a symmetrical bandwidth of B [Hz] around zero center frequency, with a frequency sweep rate $d_{f}$ [Hz/s], can be defined as [30]

(2) $$r_{B,d_{f}}\left[t\right]=\left\{\begin{array}{cc} \exp\left(2\pi j\left(-\frac{B}{2}sgn\left(d_{f}\right)+\frac{d_{f}}{2}t\right)t\right) & t\in \left[0,\ldots ,\frac{B}{d_{f}}\right]\\ 0 & otherwise \end{array}\right.$$

Therefore, if the signal sampling rate is $f_{s}$ [Hz] and the expected chirp frequency change rate is $d_{f}$ [Hz/s], the template signal for correlation has a duration time $f_{s}/d_{f}$ [s] and contains $f_{s}^{2}/d_{f}$ samples of signal with instantaneous frequency starting from $-f_{s}/2$ when $d_{f}$ is positive and from $+f_{s}/2$ for a negative frequency change rate. The normalized cross-correlation of received signal $x\left[n\right]$ with the chirp template is therefore defined as [31]

(3) $$R_{I}\left[t\right]=\frac{\left|\sum_{\tau =0}^{f_{s}/d_{f}}x\left[t+\tau \right]\cdot r_{f_{s},d_{f}}{\left[\tau \right]}^{*}\right|}{\sqrt{\sum_{\tau =0}^{f_{s}/d_{f}}x{\left[t+\tau \right]}^{2}}\cdot \sqrt{\sum_{\tau =0}^{f_{s}/d_{f}}{\left|r_{f_{s},d_{f}}\left[\tau \right]\right|}^{2}}}$$

**Figure 4:**
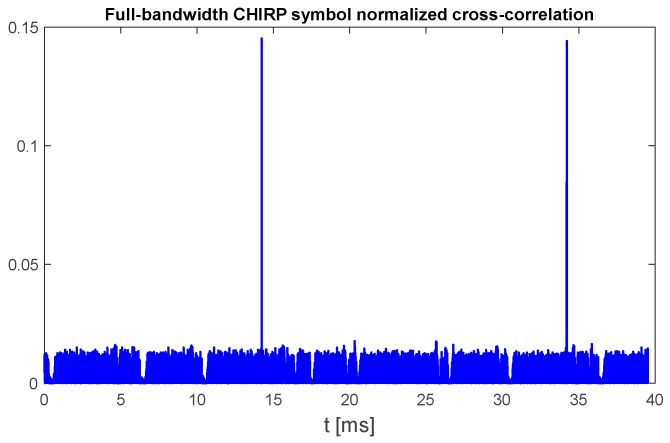
Normalized cross-correlation of UAV emission with full-bandwidth template chirp symbol (125 MHz sweep range). UAV model: DJI AIR 2S, 18 MHz signal bandwidth, sampling rate 125 MHz, template signal duration time 450 μs.

An example plot in Figure 4 shows the results obtained for the same UAV signal fragment duration, 40 ms, that was used to prepare Figure 3. As it can be noticed, this signal contains only two up-chirp signals.

**Figure 5:**
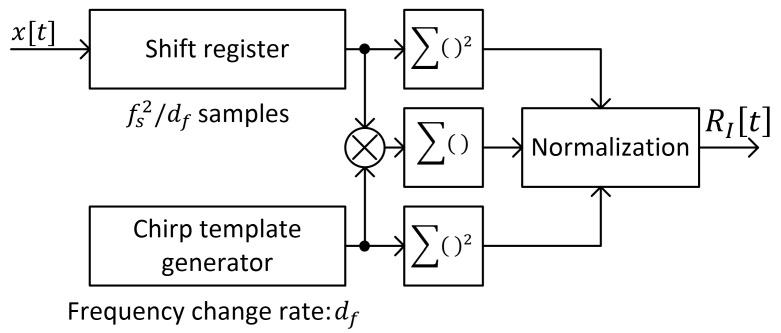
Block diagram of chirp symbol detection algorithm based on correlation with full-bandwidth chirp template (method I).

A straightforward implementation of full-bandwidth chirp correlation leads to the algorithm with the block diagram presented in Figure 5.

The presented figure shows that such an approach allows us to obtain results comparable to the correlation with the exact shape of the chirp symbol, but some significant differences must be taken into account:

- The expected maximum of the cross-correlation function of the signal with bandwidth B and the full-bandwidth chirp template, whose bandwidth is limited by the range of monitored frequencies (signal sampling rate $f_{s}$), is $B/f_{s}$.
- When the noise level is negligible compared to the UAV signal power level (i.e., high signal-to-noise ratio), the ratio between peaks caused by the presence of chirp symbols and unwanted correlation components caused by the presence of non-chirp symbols remains similar to the case with band-limited correlation, but the presence of high-power noise or the presence of other signals in the monitored band increases the level of unwanted correlation peaks and may increase the probability of false detection.
- An unknown difference between the center frequency of the UAV signal and the template signal center frequency (zero frequency in baseband in case of symmetric template start and stop frequencies) causes an unknown time shift between the position of peaks in the correlation plot and the real occurrence of chirp symbols, which is related to frequency difference by the value of the frequency rate change.

Moreover, in the case of 36 MHz UAV signal bandwidth, when chirp symbols contain two components transmitted simultaneously with approx. 18 MHz shift in frequency between them, cross-correlation of such a signal with a single-sweep chirp template results in two peaks with approximate delay between them close to the symbol duration time (approx. 66 μs).

The chirp template $r_{f_{s},d_{f}}\left[\tau \right]$ covers the whole frequency band defined by the sampling frequency $f_{s}$; therefore, as long as the UAV signal spectrum is properly sampled (whole signal spectrum is between $-f_{s}/2$ and $f_{s}/2$ relative to center frequency of signal recording), the correlation function $R_{I}$ should indicate the presence of chirp symbols independently from the UAV signal carrier frequency. Moreover, changing the signal frequency by some arbitrary frequency shift $\Delta_{f}$ results in the following changes in the numerator of (3):

(4) $$x\left[t+\tau \right]\cdot \exp\left(2\pi j\Delta_{f}\left(t+\tau \right)\right)\cdot \exp\left(-2\pi j\left(-\frac{f_{s}}{2}sgn\left(d_{f}\right)+\frac{\tau d_{f}}{2}\right)\tau \right)=\;=x\left[t+\tau \right]\cdot \exp\left(2\pi jt+2\pi j\left(-\frac{f_{s}}{2}sgn\left(d_{f}\right)+\frac{\tau d_{f}}{2}-\Delta_{f}\right)\tau \right)=\;=x\left[t+\tau \right]\cdot \exp\left(2\pi jt+2\pi j\left(\frac{-f_{s}}{2}sgn\left(d_{f}\right)+\frac{\left(\tau -\frac{\Delta_{f}}{d_{f}}\right)d_{f}}{2}\right)\left(\tau -\frac{\Delta_{f}}{d_{f}}\right)+\varphi_{0}\right)$$

where $\varphi_{0}$ is the constant phase shift that does not influence results of (3). Therefore, change in the frequency of received signal $x\left[t\right]$ by $\Delta_{f}$ is equivalent to performing a correlation of the signal at nominal frequency but with an additional time shift equal to $\Delta_{f}/d_{f}$, as long as both the whole spectrum of the signal before the frequency shift and after the frequency shift is inside the range $-f_{s}/2$ to $f_{s}/2$. Thus, the performance of the proposed chirp detection method does not depend on signal center frequency.

### 3.2. Method II

The next method is based on the properties of discrete Fourier transform of complex signals. The chirp symbol to be detected starts from the frequency $f_{L}$ and ends at $f_{H}$ (or vice versa in case of down-chirp symbols), but only the signal bandwidth $B=f_{H}-f_{L}$ is known while the center frequency $f_{c}=\left(f_{H}+f_{L}\right)/2$ is unknown. But when samples of signal $x\left[t\right]$ are reordered in reverse sequence (time reversal) $x\left[T_{c}-t\right]$, where $T_{c}$ is chirp symbol duration time, the spectrum is mirrored [32]; thus, the chirp signal starts with frequency $-f_{L}$ and ends at $-f_{H}$. The multiplication of chirp signal samples in normal and reverse order creates another chirp signal with doubled bandwidth (2B) and doubled frequency rate change $\left(2d_{f}\right)$ but with zero center frequency. Therefore, the chirp signal at an arbitrary center frequency $f_{c}$ is converted to another signal with all parameters, start frequency $\left(-B\right)$, stop frequency $\left(+B\right)$, and frequency rate change $\left(2d_{f}\right)$, defined, so simple normalized cross-correlation with a proper chirp template $r\left[t\right]$ can be used to reveal chirp symbol presence:

(5) $$R_{II}\left[t\right]=\frac{\left|\sum_{\tau =0}^{T_{c}}x\left[t+\tau \right]\cdot x\left[t+T_{c}-\tau \right]\cdot r_{2B,2d_{f}}{\left[\tau \right]}^{*}\right|}{\sum_{\tau =0}^{T_{c}}{\left|x\left[t+\tau \right]\right|}^{2}\cdot \sqrt{\sum_{\tau =0}^{T_{c}}{\left|r_{2B,2d_{f}}\left[\tau \right]\right|}^{2}}}$$

**Figure 6:**
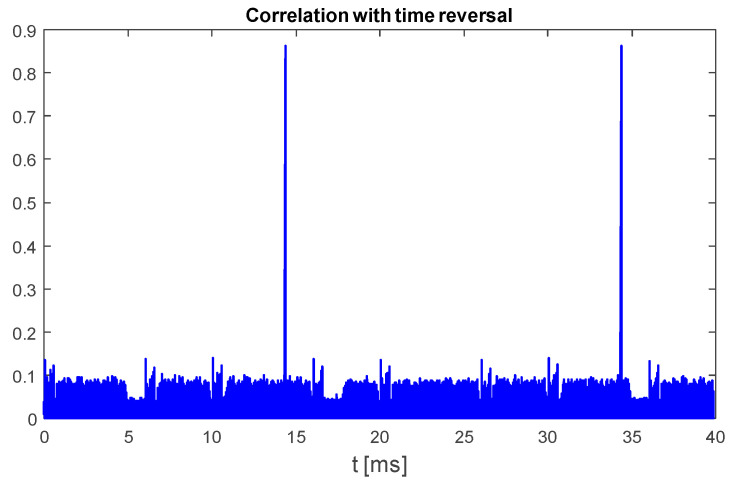
Normalized cross-correlation of time-reversed UAV emission with zero center frequency double-rate template chirp symbol. UAV model: DJI AIR 2S, 18 MHz signal bandwidth.

This method is based on the assumption that only chirp-like symbols have a time–frequency characteristic with odd symmetry around the symbol center, both in the time and frequency domains. An example plot in Figure 6 confirms that, indeed, other parts of recorded UAV communication of DJI drones do not achieve as high values of (5) as chirp symbols.

**Figure 7:**
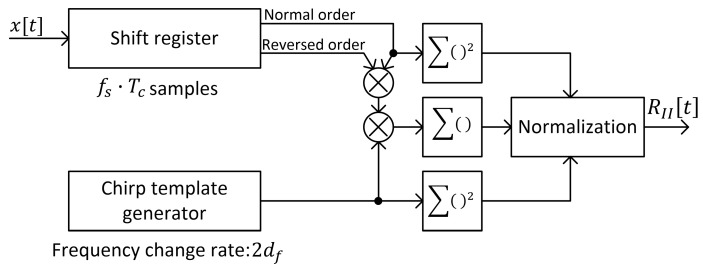
Block diagram of chirp symbol detection algorithm based on correlation with time reversal (method II).

A block diagram of a possible implementation of the algorithm that allows calculating signal correlation with time reversal according to the second method is presented in Figure 7.

Also, this chirp detection method works the same for any signal center frequency, as long as whole UAV signal spectrum is correctly sampled without aliasing in range from $-f_{s}/2$ to $f_{s}/2$. By changing frequency of signal x[t] by $\Delta_{f}$ we obtain the following change inside discrete integration in numerator of fraction (5):

(6) $$x\left[t+\tau \right]\cdot \exp\left(2\pi j\Delta_{f}\left(t+\tau \right)\right)\cdot x\left[t+T_{c}-\tau \right]\cdot \exp\left(2\pi j\Delta_{f}\left(t+T_{c}-\tau \right)\right)=\;=x\left[t+\tau \right]\cdot x\left[t+T_{c}-\tau \right]\cdot \exp\left(2\pi j\Delta_{f}\left(2t+T_{c}\right)\right)$$

However, inside integration, the time t is constant; therefore, the complex exponential in (6) only causes a constant phase shift, which is meaningless as only the modulus of the fraction numerator in (5) is important. The same rules apply for the denominator. Therefore, the shape of correlation function $R_{II}$ does not depend on UAV signal center frequency.

### 3.3. Method III

The last method of chirp symbol detection involves dividing the signal into two parts in the time domain. In the case of a chirp signal, both symbol parts have a spectrum of the same shape but with a systematic frequency shift resulting from the rate of frequency change and the time difference of the beginning of both fragments. Therefore, it is possible to correlate the first part (half) of chirp symbol with the second part after spectrum shift, which can be accomplished in time domain by multiplication with a complex sinusoid.

For a chirp symbol duration time $T_{c}$ and frequency change rate $d_{f}$, the autocorrelation function can be defined as

(7) $$R_{III}\left[t\right]=\frac{\left|\sum_{\tau =0}^{T_{c}/2}x\left[t+\tau \right]\cdot x{\left[t+\tau +\frac{T_{c}}{2}\right]}^{*}\exp\left(-2\pi jd_{f}\frac{T_{c}}{2}\tau \right)\right|}{\frac{1}{2}\sum_{\tau =0}^{T_{c}}{\left|x\left[t+\tau \right]\right|}^{2}}$$

**Figure 8:**
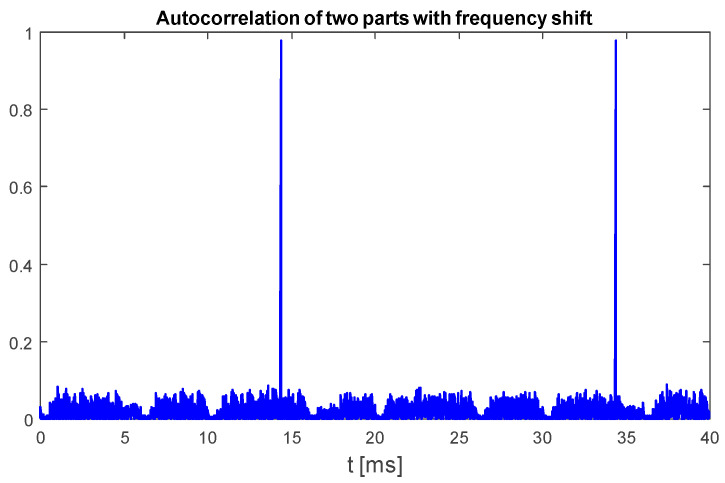
Normalized autocorrelation of UAV signal with the same signal delayed by 32 μs and shifted in frequency by 8.64 MHz. UAV model: DJI AIR 2S, 18 MHz signal bandwidth.

This method relies on a low probability of similarities in the shifted spectrum of signals other than chirp symbols. An example plot in Figure 8 shows that OFDM-based UAV communication indeed does not reveal such characteristics between chirp symbols.

**Figure 9:**
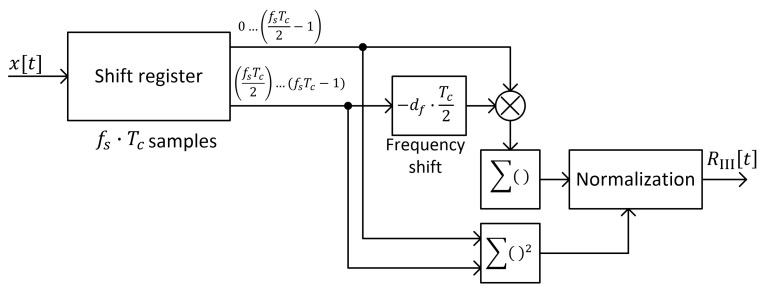
Block diagram of chirp symbol detection algorithm based on correlation with frequency shift (method III).

Signal autocorrelation with frequency shift can be obtained using an algorithm that works according to the block diagram presented in Figure 9.

It can be shown from the properties of complex frequency shifting and complex conjugation [32] that this method also does not depend on signal center frequency. The expression in discrete integration in (7) for a UAV signal x[t] with a frequency shift $\Delta_{f}$ can be simplified using

(8) $$x\left[t+\tau \right]\cdot \exp\left(2\pi j\Delta_{f}\tau \right)\cdot x{\left[t+\tau +\frac{T_{c}}{2}\right]}^{*}\cdot \exp\left(-2\pi j\Delta_{f}\tau \right)\cdot \exp\left(-2\pi jd_{f}\frac{T_{c}}{2}\tau \right)=\;=x\left[t+\tau \right]\cdot x{\left[t+\tau +\frac{T_{c}}{2}\right]}^{*}\cdot \exp\left(-2\pi jd_{f}\frac{T_{c}}{2}\tau \right)$$

which gives the same results for any arbitrary signal frequency shift $\Delta_{f}$.

### 3.4. Computational Complexity Analysis

Assume that discrete-time UAV signals consist of N samples taken at a sampling frequency $f_{s}$. The chirp frequency change rate $d_{f}$ and bandwidth B is also known. Additionally, in the case of method II and III, the symbol duration time $T_{c}$ should also be provided. All proposed chirp detection methods are based on signal processing in the physical layer; thus, a number of operations per one signal sample is a good indication of their computational complexity. Further processing of correlation results, which may include threshold detection, averaging 20 ms long fragments, or frequency domain analysis of correlation peaks will not be considered because the method of use in the proposed solutions in larger UAV detection or spectrum monitoring applications may be variable.

#### 3.4.1. Method I

In the case of method I, the full-bandwidth chirp symbol that covers whole frequency band from $-f_{s}/2$ to $f_{s}/2$ with frequency sweep rate $d_{f}$ consists of $f_{s}^{2}/d_{f}$ samples. Normalized correlation of received UAV signal with full-bandwidth chirp template requires the following:

- $f_{s}^{2}/d_{f}$ multiplications of the received signal samples with the template;
- $f_{s}^{2}/d_{f}$ squares of the received signal—however, only one squared sample is new in each step;
- $f_{s}^{2}/d_{f}$ squares of the template, which is fixed—thus, these values can be calculated only once.

Therefore, we obtain $1+f_{s}^{2}/d_{f}$ complex multiplications per one signal sample in a straightforward implementation of Equation (3), so for any arbitrary signal length of N samples, the total number of required operations depends linearly on N, which gives the algorithm a computational complexity of O(N). Further optimization is possible in case of FFT-based correlation.

#### 3.4.2. Method II

In the case of method II, signal duration $T_{c}$ corresponds to $T_{c}\cdot f_{p}$ discrete signal samples. Therefore, calculation of $R_{II}$ defined in Equation (5) requires the following:

- $2T_{c}\cdot f_{s}$ complex multiplications in the numerator for each new signal sample;
- $2T_{c}\cdot f_{s}$ complex squares in the denominator, but only one new square must be calculated with each new sample—the rest of the values are already available from previous iterations or they are constant.

**Figure 10:**
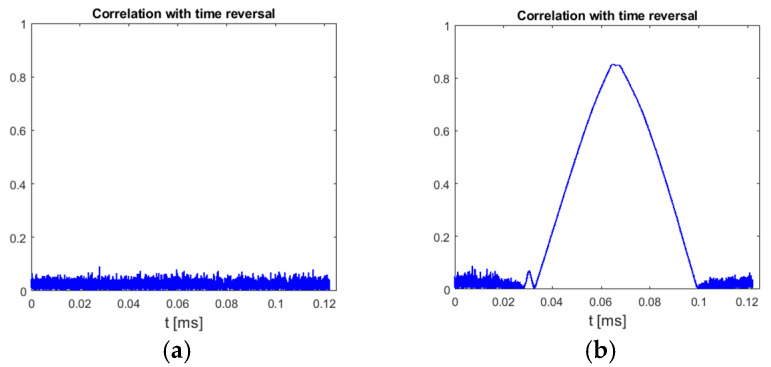
Results of processing files from CardRF dataset without (**a**) and with (**b**) chirp symbol. UAV model: DJI Mavic Pro, video transmission, method II. Signal bandwidth: 18 MHz.

The number of complex multiplications per one signal sample is $1+2T_{c}\cdot f_{s}$, so for any arbitrary signal length of N samples, the total number of required operations depends linearly on N, which gives the algorithm a computational complexity of O(N). However, by looking at Figure 10, it can be seen that the duration time of the peak that indicates the presence of a chirp symbol is comparable to the symbol duration time, which is over 66 μs for all tested DJI drones. Therefore, it is not necessary to calculate the values of (5) with each new signal sample, which allows for a large reduction in the total number of performed mathematical operations.

#### 3.4.3. Method III

In the case of method III, signal duration $T_{c}$ corresponds to $T_{c}\cdot f_{p}$ discrete signal samples, but the symbol time is divided into the first half, which remains unchanged, and the second half, whose frequency is shifted by complex multiplication. Therefore, calculation of $R_{III}$ defined in Equation (7) requires the following:

- One complex multiplication per signal sample for the frequency shift;
- $T_{c}\cdot f_{s}/2$ complex multiplications in the numerator for each new signal sample;
- $T_{c}\cdot f_{s}/2$ complex squares in the denominator, but only one new square must be calculated with each new sample—the rest of the values are already available from previous iterations.

The number of complex multiplications per one signal sample is $2+T_{c}\cdot f_{s}/2$, so for any arbitrary signal length of N samples, the total number of required operations depends linearly on N, which gives the algorithm a computational complexity of O(N). However, also in this method, the duration time of the peak that indicates the presence of a chirp symbol is comparable to the symbol duration time, which is over 66 μs for all tested DJI drones. Therefore, it is not necessary to calculate the values of (7) with each new signal sample, which allows for a large reduction in the total number of performed mathematical operations.

## 4. Verification

**Figure 11:**
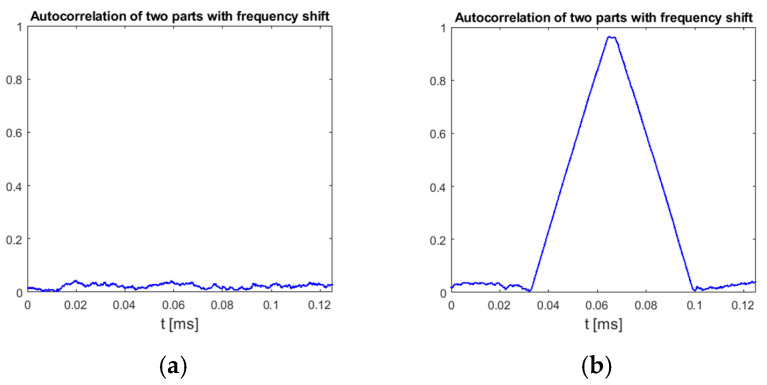
Results of processing files from CardRF dataset without (**a**) and with (**b**) chirp symbol. UAV model: DJI Mavic Pro, video transmission, method III. Signal bandwidth: 18 MHz.

The charts presented in Section 3 were prepared using signals recorded by the authors, but the detection correctness of chirp symbols in UAV signals was verified using signal samples from publicly available datasets to enable easy reproduction of results. The previously mentioned dataset “CardRF” [18] contains samples of signals from four DJI drones. Signals stored in this dataset were recorded using a digital oscilloscope connected to an antenna with a preamplifier and 2.4 GHz bandpass filter, without frequency conversion or signal preprocessing. Some details regarding the method of UAV signal recording used to prepare CardRF dataset can be found in [33]. Signals from the CardRF dataset were preprocessed by performing 2.45 GHz downconversion to obtain baseband complex samples (real signal center frequency was not estimated nor taken into account at any stage of signal processing), and then low-pass filtering with a cutoff frequency of 50 MHz and decimation were applied to obtain a sampling rate of 100 MHz. Such converted samples allowed calculating $R_{I}$, $R_{II}$, and $R_{III}$ as defined in Section 3. All signal preprocessing and calculation of correlation functions from all proposed methods was performed using Matlab scripts (Matlab version R2022a with Signal Processing toolbox, Mathworks, Natick, MA, USA). Unfortunately, due to very high sampling rate (20 GHz) caused by direct sampling of radio frequency signals, files from the CardRF dataset contain samples from a very short time period of only 250 μs, and subsequent files are not synchronized with each other (unknown part of signal is missing between parts stored in next files). For all the proposed chirp detection methods, a signal part at least 66 μs long must be available in the processed file for correct chirp symbol detection, but as these symbols are repeated once per 20 ms, the probability of the presence of a full chirp symbol in a 250 μs long file recorded starting from a random time moment is lower than 0.9%. Additionally, some files from this dataset were prepared incorrectly because when they were recorded, the analog-to-digital converter in the oscilloscope used to record the signals was overdriven. But despite these issues, a clear indication of the presence of chirp symbols was obtained in several files from the measurement series for the “Mavic Pro” drone, which is a high-quality UAV for more demanding users, also produced by DJI. Examples of results obtained for a file without a chirp symbol and a file with a chirp symbol are presented in Figure 10 (method II) and Figure 11 (method III).

**Figure 12:**
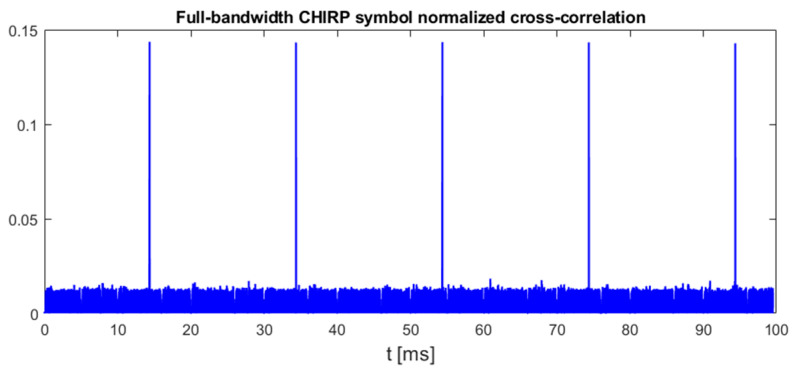
Results of processing files from DroneRF dataset for DJI Matrice 300 video transmission, method I. Signal bandwidth: 18 MHz, chirp rate: +270.2 kHz/μs.

**Figure 13:**
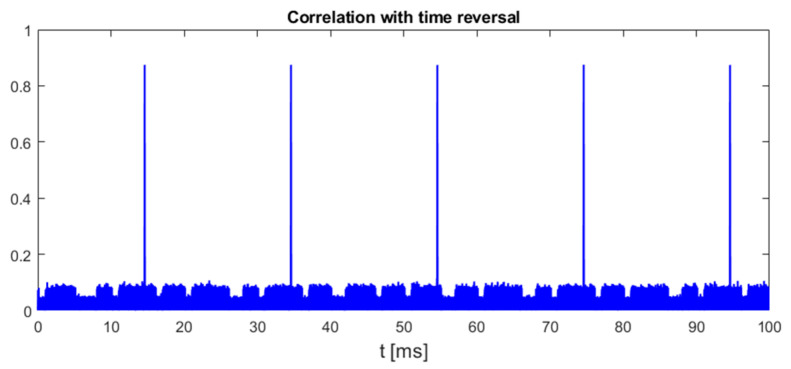
Results of processing files from DroneRF dataset for DJI Matrice 300 video transmission, method II. Signal bandwidth: 18 MHz, chirp rate: +270.2 kHz/μs.

**Figure 14:**
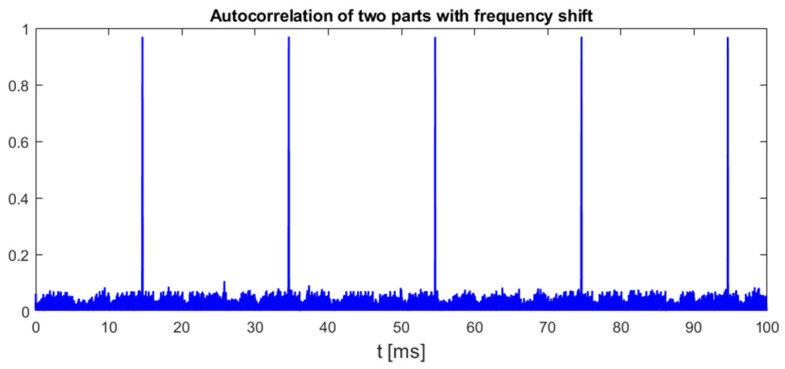
Results of processing files from DroneRF dataset for DJI Matrice 300 video transmission, method III. Signal bandwidth: 18 MHz, chirp rate: +270.2 kHz/μs.

A signal recording method that is more suitable for the described research was used in the preparation of another signal dataset, called “DroneRF” [34]. This dataset contains signals from several drones including DJI, recorded in the 2.4 GHz band using USRP X310 with a sampling rate of 100 MHz. Signal processing in X310 includes the conversion of RF real signals to baseband complex signals, filtration, and sampling, which are typical operations performed in digital receivers. Therefore, signal samples from DroneRF do not require any additional preprocessing and can be directly used to calculate $R_{I}$, $R_{II}$, and $R_{III}$ using the equations defined in Section 3. Each single file in the DroneRF dataset contains complex samples from a 1 s long time period. Taking into account the 20 ms period of chirp symbol repetition, all proposed methods should indicate the presence of 50 symbols in each file. The following Figure 12, Figure 13 and Figure 14 show example results of chirp symbol detection using all proposed methods for DJI Matrice 300 signals. For better readability, the presented results are limited to a 100 ms long fragment of UAV emission that contains five chirp symbols.

**Figure 15:**
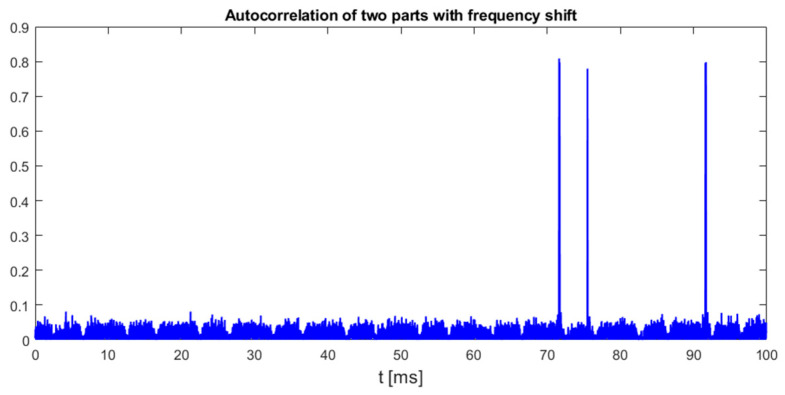
Results of processing files from DroneRF dataset for DJI Matrice 300 drone identification transmission, method III. Signal bandwidth: 9 MHz, chirp rate: −130.2 kHz/μs.

In addition to regular transmission of video stream packets from UAVs, some files in the DroneRF dataset also contain irregular signals that are most likely drone identification packets (DroneIDs), whose structure has been revealed, e.g., in publications [26,27]. But comparing this part of the transmission with the regular transmission of chirp symbols in the video signal from the UAV, the chirp symbols in the drone identification packets are transmitted irregularly and are characterized by a linear decay of the instantaneous frequency. Figure 15 presents detected down-chirp symbols in DroneID transmission for DJI Matrice 300 in the same time scale as was used in Figure 12, Figure 13 and Figure 14. Figure 15 was prepared using method III based on autocorrelation of two consequent fragments of signal with a frequency shift, but the other two methods returned very similar plots, as all of them can work with any distribution of chirp symbols in processed signals.

It should be emphasized that all three pulses clearly visible in Figure 15 were transmitted by a DJI UAV at different carrier frequencies selected randomly in the range of 2.42 GHz to 2.44 GHz, but as all the proposed chirp detection methods are not frequency-selective, all symbols were detected without the need for carrier frequency estimation.

**Figure 16:**
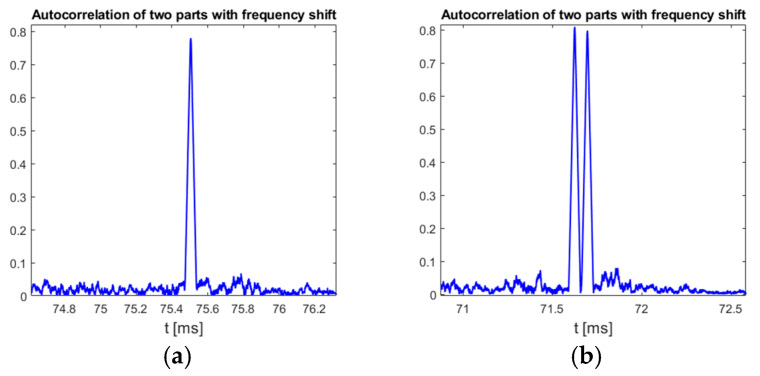
Single (**a**) and double (**b**) down-chirp symbol detection in DJI Matrice 300 drone identification transmission, method III. Signal bandwidth: 9 MHz, chirp rate: −130.2 kHz/μs.

Figure 15 also confirms that irregular emission of chirp symbols does not affect any of the proposed detection methods, as all three of them processes signals only within a time period of one chirp symbol, which is slightly more than 66 microseconds (method II and III) or a time period defined by the signal sampling rate (directly related to reception bandwidth) and chirp frequency change rate (method I) that is for sure longer than the single symbol duration but still does not exceed hundreds of microseconds. Therefore, the minimum duration time of a signal that must be processed for chirp detection is also limited to tens or hundreds of microseconds and any two chirp symbols, transmitted at any carrier frequency, with a time shift no shorter than the detector memory, can be detected independently. This feature also allows us to show that some of the down-chirp symbols transmitted from the UAV are doubled, as it is visible in the plots in Figure 16.

The presented results confirm the ability to detect the presence of chirp symbols without the need to estimate signal center frequency. The only parameter critical for correct chirp symbol detection is the signal instantaneous frequency sweep ratio, and in the case of methods II and III, it is also beneficial to know the chirp symbol duration time. Low requirements for information on signal parameters are the main advantage of the proposed signal processing methods.

**Figure 17:**
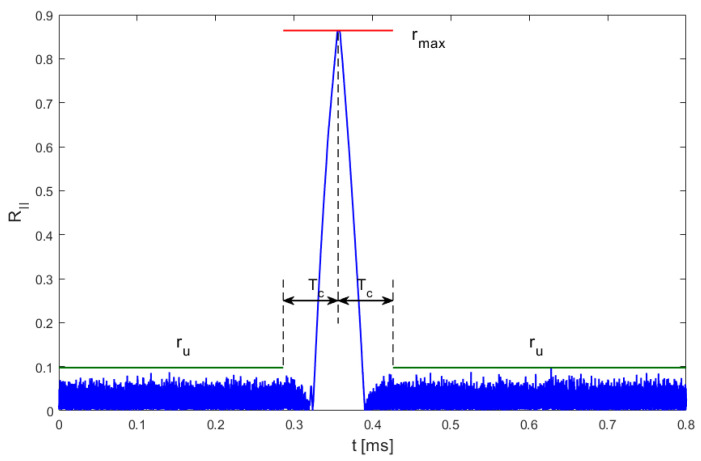
Definition of valid correlation peak ($r_{max}$) and maximal unwanted correlation value ($r_{u}$).

All the proposed chirp detection methods are based on some variants of signal autocorrelation or cross-correlation; therefore, as it is typical in all correlation-based signal detection methods, their performance depends on signal-to-noise ratio (SNR). The signals available in the DroneRF dataset were recorded in good propagation conditions and are characterized by a quite high SNR exceeding 30 dB. In real conditions, such an SNR is possible mostly in the case of direct visibility between the UAV and the receiver. Worse propagation scenarios will most likely not allow one to obtain such clearly visible correlation peaks as those presented in the exemplary charts in Figure 4, Figure 6, and Figure 8. Evaluation of chirp detection performance in variable conditions can be based on the ratio between the correlation peak value ($r_{max}$), calculated as the maximum value of (3), (5), or (7), respectively, in the analyzed time period, which indicates the true position of the chirp symbol, and as the maximum value of unwanted correlation products ($r_{u}$) that do not indicate chirp presence, calculated as the maximum from (3), (5), or (7), but only from samples that are not closer to the global peak $r_{max}$ than the chirp symbol time $T_{c}$. A graphic interpretation of these values is presented in Figure 17.

**Figure 18:**
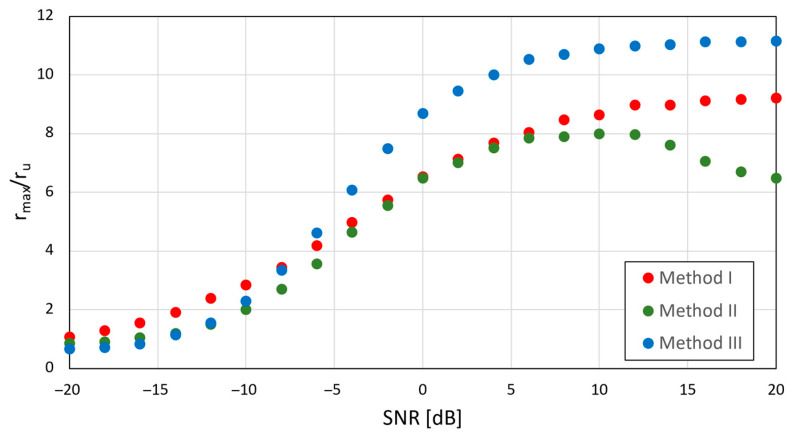
Ratio between correlation peaks ($r_{max}$) and unwanted correlation results ($r_{u}$) as a function of signal-to-noise ratio for all proposed chirp detection methods. Sampling rate $f_{s}=125$ MHz, UAV model: DJI AIR 2S, signal bandwidth: 18 MHz.

When data from open datasets are used, it is not possible to control the conditions in which these signals were recorded. A series of simulations were performed based on DroneRF files, in which the SNR was controlled by adding Gaussian noise with properly adjusted power. The DJI AIR 2S video transmission signal was selected for the test. Regular repetition of chirp symbols in this transmission allowed us to divide whole signal into 20 ms long fragments and calculate $r_{max}/r_{u}$ separately in each fragment that contains a chirp symbol. The results presented in Figure 18 are averaged values obtained by processing the whole AIR 2S transmission in the DroneRF dataset.

Values of $r_{max}/r_{u}$ below 1 mean that it is not possible to find the chirp symbol position by looking for the maximum value of $R_{I}$, $R_{II}$, or $R_{III}$, respectively. Values higher than 1 mean that the highest peak in the correlation function indicates the position of a chirp symbol, and a higher ratio of $r_{max}/r_{u}$ results in a more clear indication of chirp presence. Reliable indication of chirp presence is achievable when $r_{max}/r_{u}$ exceeds 2…3, which corresponds to an SNR greater than −13 dB for method I and −10 dB for methods II and III. However, it must be noted that the presented values depend on the signal sampling frequency and UAV signal bandwidth, because wideband noise occupies the whole frequency range defined by sampling frequency $f_{s}$, not only the range of frequencies used by the UAV for signal emission.

Further signal processing can be based on simple threshold detection, valid for both regular and irregular chirp symbol emission, or more advanced analysis of regular pulse stream in frequency domain. However, the decision-making rules and method of integration of the proposed chirp detection methods are application-dependent and will not be discussed.

## 5. Conclusions

The proposed chirp detection methods will rather not be used as standalone methods of UAV emission detection, because as far as now, chirp symbols (OFDM symbols with Zadoff–Chu sequences that are characterized by linear frequency sweep in time) were found only in emissions from DJI drones that use the OcuSync protocol, and although this company’s products constitute a significant part of the global UAV market, drones from other manufacturers are also popular. Nevertheless, fast and reliable detection of DJI transmission can be helpful in solutions that require some kind of UAV classification and in hybrid systems that combine data from many sensors using various drone detection methods to achieve higher detection quality and efficiency. Compared to classic solutions based on the correlation of the received signals with a locally generated reference signal, or more sophisticated methods based on protocol decoding, the proposed chirp detection methods do not require the signal center frequency to be known or estimated prior to correlation, which simplifies the implementation of the proposed method. In fact, all plots based on files from the “DroneRF” dataset were obtained by processing the signal samples by Matlab scripts with straightforward implementation of the proposed methods I, II, and III, without any additional signal preprocessing such as filtration, frequency estimation, or correction. Variable signal center frequency, observed between files and even within single file, was not analyzed or compensated in any way. Future work should focus on two directions. First, research should expand to include signals from a larger number of drones, including devices from manufacturers other than DJI. Second, decision-making algorithms remain to be developed that will make UAV detection decisions based on correlation results defined in the proposed chirp signal detection methods. The decision-making process will depend on many factors, such as the system design (whether chirp detection operates independently or in conjunction with other signal processing methods), the conditions under which UAV signals will be received, and the required detector response time.

## Data Availability

Presented results were obtained using public UAV signal datasets listed in References.
